# The Impact of the COVID-19 Pandemic on Poles’ Nutritional and Health Behaviour and Quality of Life—A Pilot Study

**DOI:** 10.3390/ijerph182010656

**Published:** 2021-10-12

**Authors:** Ewa Raczkowska, Dominika Mazurkiewicz, Jagoda Ambrozik-Haba, Michaela Godyla-Jabłoński

**Affiliations:** Department of Human Nutrition, Faculty of Biotechnology and Food Science, Wrocław University of Environmental and Life Sciences, ul. Chełmońskiego 37, 51-630 Wrocław, Poland; ewa.raczkowska@upwr.edu.pl (E.R.); jagoda.ambrozik-haba@upwr.edu.pl (J.A.-H.); michaela.godyla@upwr.edu.pl (M.G.-J.)

**Keywords:** pandemic, COVID-19, lifestyle, quality of life, nutritional behaviour, health

## Abstract

The coronavirus (COVID-19) pandemic, which has lasted for over a year, has affected everyone’s lives. It is interesting to examine how populations cope with the new situation and to learn about the impact of the epidemic on quality of life. The aim of this study was to assess the impact of the COVID-19 pandemic on changes in selected elements of lifestyle compared to the pre-pandemic period among adult Polish residents. The impact of the COVID-19 pandemic on selected features of quality of life was examined. It was found that, for some distinguishing factors (i.e., quality of life, health status and sleep quality), there were no statistically significant differences. Other distinguishing factors (i.e., ability to perform daily living activities, ability to work and personal relationships) were statistically different. Another part of the study was to assess changes in the frequency of consumption of specific food groups. The vast majority of respondents declared no changes in the frequency of consumption of selected food products. The majority of respondents declared that the COVID-19 pandemic did not cause a change in the frequency of drug use (i.e., cigarettes, alcohol and coffee).

## 1. Introduction

The emergence of a new virus, COVID-19, in the world has caused global paralysis. The SARS-CoV-2 virus was first identified in December 2019 in Wuhan, China. The pace of the spread of this virus beyond the Asian continent was very fast, and, as a consequence, the World Health Organization (WHO) declared a pandemic on 11 March 2020 [1,2]. It was connected with the introduction of the so-called lockdown (i.e., limiting contacts and movement of people to a minimum). The outbreak of the pandemic had an impact not only on health and health care but also on the mental well-being, economic status and lifestyle of people around the world. The pandemic changed people’s health, eating and physical activity behaviour. Weight gain was found to be related to the change in diet and a sedentary lifestyle [2,3]. Long-term limitation of interpersonal contacts or isolation at home can cause chronic stress, depressed mood and even depression. These factors contribute to an increased sense of hunger and reaching for products that improve mood, are rich in simple sugars and saturated fatty acids (sweets, salty snacks, sweetened carbonated drinks and fast food) or drugs (alcohol and cigarettes) [4,5,6]. A sudden change of lifestyle and social isolation related to a constant staying in one place (usually one’s place of residence) resulted in a compulsory change to digital education, limitation of physical activity and staying outdoors in favour of a sedentary lifestyle, mainly in front of a computer or TV. The monotony of everyday life and staying in one place, regardless of the activity performed, may additionally increase the feeling of fatigue, reduced ability to work or snacking on extra meals or products [7]. A long separation from society may also foster a more frequent search for so-called normality, which may be manifested in a more frequent ordering of ready meals and the willingness to support gastronomy [3]. Another important aspect of dietary change during the pandemic was the restricted access to food due to some stores closing, restricted movement and restricted access to fresh food (including fruits, vegetables and fresh fish) in favour of processed and finished products. The development of the pandemic also had an impact on the economic status by lowering wages or loss of jobs, which significantly reduced the quantity and quality of purchased products and meals [8,9].

The aim of the study was to assess the impact of the COVID-19 pandemic on the change of selected lifestyle elements in comparison to the time before the pandemic among adults in Poland. The study comprised respondents’ subjective assessments of whether and to what extent selected eating behaviours have changed.

## 2. Materials and Methods

### 2.1. Studied Population

The study was conducted in 2021, and the research group consisted of 174 people. The characteristics of the study sample, including its sociodemographic characteristics, are presented in Table 1.

The survey was conducted from 15 to 29 April 2021. The criteria for inclusion in the study were: age ≥ 18 years of age; Polish nationality; female or male gender. The questionnaire was created using a Google form that was accessible from any device with an Internet connection. The survey was disseminated via social networks (especially Facebook). This method of data collection can make it impossible to fully control the population parameters (as in the case of probabilistic sampling). In this study, however, this method was effective in achieving the assumed goal, as it allowed for the widespread dissemination of the questionnaire during the context of the COVID-19 pandemic, when direct contact was impossible and, in addition, when territorial limitations existed. Furthermore, recent research results have shown that during this pandemic, Internet use has increased significantly, making it easier to reach different groups of people [10].

The study was conducted in accordance with the Helsinki Declaration [11]. Personal and participant data were anonymised in accordance with the general regulation on the protection of personal data of the European Parliament (GDPR 679/2016). The respondents did not provide their names or computer IP address. All participants were informed about the objectives of the study and its anonymity before providing informed consent to take part and complete the survey. Subsequently, each survey was uploaded to the Google platform, and the final database was downloaded to Microsoft Excel.

We received a total of 230 questionnaires, but 56 of them were not taken into account for the following reasons (31 questionnaires were filled in by people <18 years of age, 7 were filled in by people living outside Poland and 18 people did not answer all the questions). Ultimately, the responses obtained from 174 people were analysed.

### 2.2. Applied Questionnaire

An original survey questionnaire was used to obtain the necessary data. The scope of collected data included sociodemographic characteristics (gender, age, place of residence, occupational status, body weight and height), questions assessing quality of life created on the basis of the shortened questionnaire called the World Health Organization quality of life (WHOQOL)-Bref, and questions about eating habits in two periods, during the COVID-19 pandemic and before it [12]. The questionnaire was previously validated by estimating its reliability (Cronbach’s alpha = 0.78). The questionnaire was anonymous, which allowed us to obtain reliable surveys. The questionnaire used in the research is included in the supplementary Materials (Table S2).

### 2.3. Statistical Analysis

The Shapiro–Wilk test was used to assess the normality of the distribution. Results were compared using the chi-square test and Kruskal–Wallis test with rank-biserial correlation (due to non-parametric distribution). For all analyses *p* < 0.05 was assumed. The STATISTICA 13.3 PL (StataCorp LP., College Station, TX, USA) program was used to carry out the statistical analysis.

## 3. Results

The impact of the COVID-19 pandemic on selected quality of life traits was examined (Table 2). It was found that when comparing the declared levels of the distinguishing factors, during the pandemic and before the pandemic, for some items, there were no statistically significant differences (quality of life, health status and sleep quality). Other discriminators were statistically different.

Table 2 shows selected elements of quality of life as assessed by the subjects. Respondents rated their ability (vitality and energy to live) during the pandemic as worse than during the time before the pandemic. It was observed that the number of negative and neutral answers increased, while the number of positive answers decreased. When describing the state before the pandemic, the largest group of respondents to the question “How satisfied are you with your ability to perform your daily living activities?” chose the answer “satisfied” and “neither satisfied nor dissatisfied” when describing the state during the pandemic. In the case of ability (readiness and efficiency) to work, the respondents also declared a deterioration of the discussed characteristic. In the case of capacity, an increase in the number of negative and neutral answers was observed, while the number of positive answers decreased. When describing the state before the pandemic, the largest group of respondents to the question “How satisfied are you with your capacity for work?” chose the answer “satisfied” and “neither satisfied nor dissatisfied” when describing the state during the pandemic. In the assessment of the variability of interpersonal contacts, a negative impact of the pandemic on this differentiator was also observed. As in the case of the two previously discussed distinctions, the number of negative responses and neutral responses increased, while the number of positive responses decreased. When describing the state before the pandemic, the largest group of respondents to the question “How satisfied are you with your personal relationships?” chose the answer “satisfied” and “dissatisfied” when describing the state during the pandemic.

**Figure 1:**
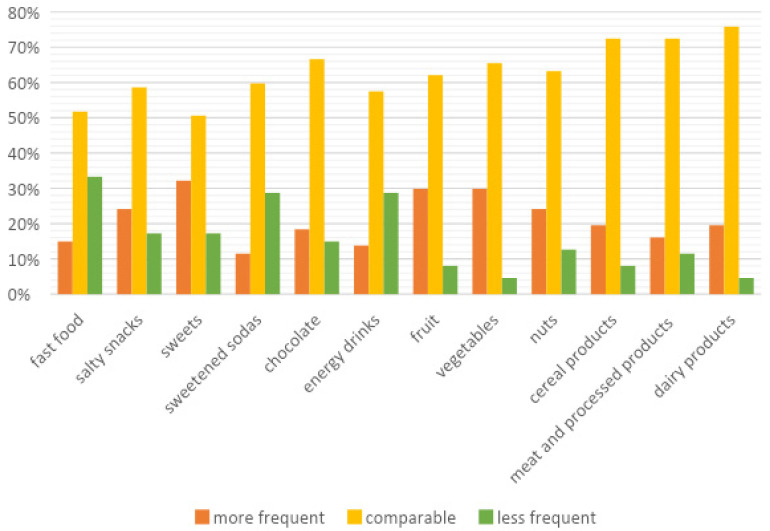
Frequency of consumption of selected food groups, comparing the time before and during the COVID-19 pandemic; (n = 174); The results are presented as a percentage of the responses obtained from the participants.

Figure 1 shows the changes in the frequency of consumption of selected groups of food products before and during the pandemic. For all food categories, the vast majority of respondents declared no change in their frequency of consumption. As for declarations of more frequent product consumption, the largest percentage of respondents declared more frequent consumption of sweets, fruits and vegetables. On the other hand, less frequent consumption of products declared by the largest group of respondents concerned fast foods, sweetened sodas and energy drinks.

The relationship between the change in frequency of consumption of selected food groups and the body mass index (BMI) and age of the respondents was also investigated (Table 3). A significant variation was found between the BMI of the respondents and the frequency of consumption of salty snacks, sweets, sweetened sodas, chocolate and cereal products. Variability was related to the declaration of less frequent consumption of a particular product during the COVID-19 pandemic and comparable frequency of consumption of the product. In the case of the relationship between the age of respondents and changes in the frequency of consumption of selected groups of food products, significant variation was noted for the following products: salty snacks (more frequent consumption vs. comparable), sweetened sodas (less frequent consumption vs. comparable), energy drinks (more frequent consumption vs. comparable and more frequent vs. less frequent consumption), fruits (less frequent consumption vs. comparable and more frequent vs. less frequent consumption), vegetables (more frequent vs. comparable consumption), nuts (less frequent vs. comparable and more frequent vs. less frequent consumption), cereals (more frequent vs. comparable consumption), meat and processed meat products (less frequent vs. comparable consumption) and dairy products (more frequent vs. comparable consumption).

It was also assessed how the respondent’s current occupation and how their change in income affected the change in frequency of ordering takeaway/self-cooking meals during the COVID-19 pandemic (compared to before the pandemic) (Table 4).

Based on the analysis, no association was found between the change in income and the change in the frequency of ordering meals. On the other hand, the association between the respondent’s current occupation and the change in the frequency of ordering meals was confirmed. In the case of people on a pension or unemployed, 100% of respondents declared a decrease in the frequency of ordering takeaway meals compared to the time before the pandemic. People with mixed employment during the pandemic declared an increase in the frequency of ordering takeaway meals compared to before the pandemic. However, about half of the students declared no changes. Based on the analysis, a relationship was found between a change in income and a change in the frequency of self-cooking. However, no relationship was confirmed between the respondent’s current occupation and a change in the frequency of self-cooked meals. People who lost their jobs during the COVID-19 pandemic answered “no” (100%) to the question “Do you prepare your own meals more often over the past year?”. People whose monthly income changed during the pandemic also mostly declared more frequent self-catering. Only in the group of respondents whose income did not change over the past year, the largest group were those who declared that during the COVID-19 pandemic they prepared meals on their own less frequently.

**Figure 2:**
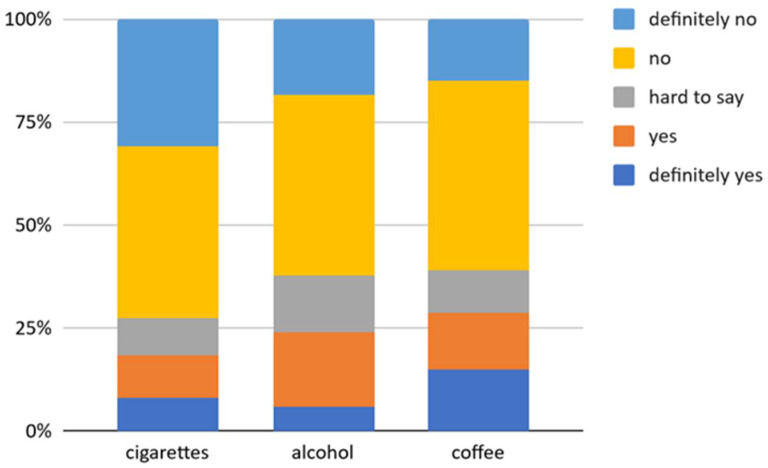
Change in frequency of use of drugs during the COVID-19 pandemic (compared to the time before the COVID-19 pandemic); (n = 174); results are presented as a percentage of responses obtained from respondents.

The questionnaire also asked about the impact of the COVID-19 pandemic on changes in the frequency of use of drugs (cigarettes, alcohol and coffee). The vast majority of respondents denied that the COVID-19 pandemic caused an increase in the frequency of drug use (Figure 2).

Relationships between the declared change in frequency of use of drugs during the COVID-19 pandemic and the respondent’s current occupation and selected characteristics describing quality of life were also examined (Table S1).

Significant correlations were found between respondents’ reported changes in smoking frequency during the COVID-19 pandemic and their occupation, ability to perform activities of daily living and ability to work. The pandemic was deemed to have had no effect on the smoking frequency of respondents belonging to groups such as unemployed, students, retirees and permanent, online and mixed workers. Almost all people who were either very satisfied or satisfied with their ability to perform daily activities reported no impact of the pandemic on their smoking frequency. As declared satisfaction with one’s own abilities decreased, the percentage also decreased of people claiming that the pandemic has had no impact on their smoking frequency.

Significant correlations were found between respondents’ declared changes in frequency of drinking alcohol during the COVID-19 pandemic and their occupation, ability to perform activities of daily living and ability to work. Regardless of their current occupation, most respondents reported that the pandemic has had no effect on their frequency of drinking alcohol. In the case of unemployed and retired people, all respondents declared that they did not consume more alcohol in the past year compared to the period before the pandemic. As declared satisfaction with one’s possibility of performing everyday activities increased, the number of people declaring that the pandemic has had no impact on their frequency of alcohol consumption also increased. On the other hand, nearly half of the people who were very dissatisfied with their ability to perform activities of daily living answered “yes” to the question “Have you drunk alcohol more often in the past year?” The relationships between the ability to work and reported changes in frequency of drinking alcohol during the COVID-19 pandemic were similar.

Significant correlations were found between respondents’ reported changes in frequency of drinking coffee during the COVID-19 pandemic and their current occupation and sleep quality. Regardless of their current occupation, the majority of respondents said the pandemic has had no effect on their frequency of drinking coffee. In the case of unemployed and retired people, all respondents reported that they did not drink more coffee in the past year compared to the period before the pandemic. Only online workers reported drinking more coffee in the past year compared to previously. As declared satisfaction with one’s ability to work during the pandemic decreased, the percentage of people declaring no impact of the pandemic on their frequency of drinking coffee also decreased. Of those who said they were very satisfied with their sleep quality during the pandemic, only a quarter reported an increase in coffee consumption over the past year. The study also found that coffee consumption increased significantly during the pandemic, and sleep satisfaction decreased among the respondents.

## 4. Discussion

The COVID-19 pandemic requires adaptation to new conditions. For this reason, it is necessary to understand how the populations of different countries deal with this global catastrophe and its impact, including with respect to quality of life, in order to effectively plan appropriate actions and assistance. The authors of this article examined the impact of COVID-19 on selected aspects of Poles’ quality of life and nutritional behaviour. The study identified changes in people’s lifestyle, choice of food products and approach to ordering takeaway or self-prepared meals. The study also identified several key consumer trends that are currently shaping eating and health habits in Poland. So far, few research results have been published assessing the impact of the coronavirus on selected lifestyle elements and nutritional behaviours among Poles.

In the authors’ research, a significant negative change in interpersonal relationships was observed during the pandemic, accompanied by decreased life vitality and productivity at work (Table 2). Zhang and Ma conducted a study assessing changes in Chinese people’s mental health and quality of life due to the COVID-19 pandemic. They found that 52.1% of the respondents were terrified and anxious as a result of the pandemic. Moreover, they observed that age had a significant influence on the respondents’ selected answers, e.g., “I feel helpless due to the COVID-19 pandemic: (*p* = 0.049), “I feel terrified of the COVID-19 pandemic” (*p* = 0.002) and “I am concerned about the COVID-19 pandemic” (*p* = 0.001). They identified no significant correlation between mental health and selected sociodemographic characteristics (e.g., gender and level of education). The authors also observed that during the pandemic, the majority of respondents experienced an increase in support from family (63.9%) and friends (64.6%), albeit this was true to a lesser extent among people aged 41–50 as compared to other age groups [13]. The pandemic has contributed to feelings of isolation due to social distancing, fear of contagion and the death of oneself and/or relatives, uncertainty about the future, limitations in travelling and access to social activities and unemployment or fear of losing one’s job, all leading to the disturbance of interpersonal relationships [14,15,16]. Hussain et al. conducted research assessing the impact of the pandemic on family relationships. Most of the respondents (52.6%) declared no changes regarding the nature of their relationships with family, parents and children during the lockdown period, while 24.9% reported improved relationships and 21.7% a reduction in family contact [17].

Another aspect discussed in this study is a comparison of respondents’ sleep quality between the period prior to the onset of the pandemic and during it. There were no significant differences in this respect (Table 2). Kocevska et al. found that the effect of COVID-19 on sleep quality varied significantly among participants and depended on their quality of sleep prior to the pandemic. In addition, approximately 25% of people with pre-pandemic insomnia experienced significant improvements in sleep quality, while 20% of those who slept well before the pandemic experienced worse sleep following the outbreak. Moreover, changes in sleep quality during the pandemic were primarily associated with increased anxiety [18]. On the other hand, Targa et al. found that the outbreak of the pandemic caused a significant deterioration in the quality of sleep (*p* = 0.035), which was significantly correlated with a negative mood (*p* = 0.002) [19]. Isolation also had a major and varied effect on physically active participants, who experienced a significant decrease (*p* < 0.05) in their levels of physical activity, sleep quality and well-being, whereas physically inactive participants did not experience significant changes [20].

The next part of the research was to evaluate changes in the frequency of consumption of various groups of food products. Most of the respondents declared no changes in the frequency of consumption of selected food products. Interestingly, around 30% of people surveyed said they consumed more fruit and vegetables during the pandemic and, at the same time, consumed less fast food, sweetened soda and energy drinks (Figure 1). Similar conclusions were drawn by Hassen et al., who demonstrated a beneficial change in eating habits during the pandemic. Consumers reduced their consumption of fast food and sweet and salty snacks. At the same time, their supply of water, fruit and vegetables increased [21].

Other studies have identified different behaviours in the United States, Italy and Canada, where consumers have proved more likely to purchase unhealthy food products, mainly related to negative emotions such as fear, panic and anxiety [22,23]. There have been many cases of panic around the world when buying foods with a long shelf life (e.g., grain products, preserved products and frozen foods). Baker et al. yielded similar conclusions [24]. A possible reason for focusing on buying groceries is a behavioural response to feelings of stress and insecurity [25]. Italian studies by Scarmozzino and Visiola assessed the effects of COVID-19 on the purchase and consumption of food products, showing that nearly 50% of respondents consumed more food during quarantine, especially sweet and salty snack products. Additionally, an increase in body weight was observed during this period. According to the respondents, such behaviour was primarily associated with increased feelings of fear [22]. Ammar et al. conducted an international study, which showed that during quarantine, eating habits changed adversely (including increased food consumption, greater snacks consumption and increased number of main meals). Such eating behaviour, along with decreased physical activity, can compromise people’s health [26]. Our own research identified that the choice of certain food products was significantly related to respondents’ body mass index (BMI) and age (Table 3). Salty snacks, sweets and sweetened drinks were less often chosen by people with a lower BMI during the pandemic. At the same time, younger people consumed more salty snacks, energy drinks and cereal products and fewer sweetened drinks, fruits and nuts. Older people paid attention to the greater supply of fruits and nuts. To our knowledge, this is one of few studies to have assessed the impact of BMI and age on the choice of specific food products during a pandemic. The choice of food products with a higher nutritional value by people with a lower BMI may be due to the fact that they are more conscious of the consequences of eating (e.g., high-energy) food, while at the same time having their physical activity limited by the pandemic. Research by Sidor and Rzymski also confirmed that snacking during quarantine was more common among overweight and obese people. In addition, obese people were characterised by the lowest frequency of consumption of fruit, vegetables and legumes, but the highest frequency of consumption of fast food, meat and dairy products. Furthermore, a positive correlation was observed between respondents’ age and consumption of meat, sweets and coffee, but a negative correlation between age and consumption of vegetables, including legumes and fruit [27]. On the basis of research by Baker et al. carried out in the United States, a significant reduction in expenditure in restaurants and retail was demonstrated. This was largely related to territorial limitations. At the same time, the authors identified an increase in spending on food supplies for at home, which was associated with more frequent self-preparation of meals in household gas [24]. Over the year, Internet sales in the United States increased by 233% [28]. The authors also observed a change in ordering takeaway meals. They identified a significant relationship between respondents’ current occupation and changes in the frequency of ordering meals. Nearly 30% of people studying and working at home declared ordering takeaway meals more frequently than before. The opposite tendency was apparent among unemployed people, retirees and pensioners. Changes in income during the pandemic did not significantly affect the frequency of ordering takeaway meals (Table 4).

More home cooking was observed during the COVID-19 pandemic. This was mainly related to the closure of restaurants, but also the fear of becoming infected. This behaviour was additionally associated with more frequent planning of purchases and spending on food. One study found that nearly half of Americans prepared their own meals more often during quarantine than before the onset of the pandemic [29]. There was a significant impact of income changes during the pandemic on the frequency of self-catering. People who lost their jobs started to prepare their own meals. Furthermore, respondents whose incomes changed over the past year (whether increased or decreased) were more likely to prepare meals at home. About 44% of people whose income remained the same did not choose to cook or bake on their own (Table 4). A positive aspect of the COVID-19 pandemic was spending more time with the family, as congregating became a new form of entertainment.

During the COVID-19 pandemic, millions of people worldwide were forced to stay at home for extended periods of time. Such circumstances can generate stress and represent a challenge, especially for people who use drugs such as cigarettes, alcohol and coffee. Among the vast majority of respondents, the outbreak of the pandemic did not change their use of the above-mentioned drugs (Figure 2). Caponnetto et al. showed that the use of traditional cigarettes decreased slightly. Among a group of ex-smokers, about one-third of the respondents declared their willingness to restart smoking, while in the group of those who had never smoked, a few participants declared their intention to start [30]. Sidor and Rzymski, however, drew different conclusions. It has been shown that nearly 50% of smokers during quarantine used cigarettes more often. Additionally, people addicted to alcohol used it more often during isolation. An increase in the frequency of alcohol consumption has also been observed among smokers [3]. The factors significantly affecting changes in the amount of drugs used were the respondents’ type of work, their ability and willingness to work and undertake daily duties, and their quality of sleep. As declared satisfaction with one’s own abilities and vitality decreased, the percentage of people declaring no influence of COVID-19 on their frequency of smoking and drinking alcohol decreased. On the other hand, the only group in the majority (54.5%) who declared drinking coffee more frequently during the pandemic was people working online (Table S1). The pandemic is an event that has exacerbated the stress of having to adapt to a long stay at home. Adaptation to new conditions is usually more difficult for full-time employees than for students or unemployed people, as it is often associated with the need to switch to a remote work system, which may increase stress [31]. On the other hand, smokers often perceive cigarettes as a stress reliever [32].

The strength of our research is that it is one of few studies to have been conducted with a Polish sample. In our opinion, similar research should also be carried out in other countries in order to plan support for people affected by the pandemic at various levels of life in the best possible way for a given society. In addition, the questionnaire used was validated, guaranteeing the quality and the accuracy of the conclusions drawn. An additional advantage of this research was that it utilised a wide cross-section of society, including in terms of age (from 18 to 67 years old). However, our study was limited by the small size of the group and the lack of direct contact with respondents (due to the increased risk of infection, the survey was conducted online), making it impossible to generalise the results to the entire Polish population. Our results are of great importance for public health and health policy. Differences in health, quality of life and eating habits before and during the COVID-19 pandemic have been attributed to the pandemic itself. However, these differences may have been influenced by a number of other individual and social factors. The results of the survey may have been influenced by bias, such as social prejudice and non-response (i.e., only people who were computer literate and had access to digital devices with the Internet were included in the survey). The planned follow-up study will assess how the population is responding to the future trajectory of the COVID-19 pandemic to assess the long-term effects of the outbreak and explore resources to better cope with it. Presented here is a preliminary study to show changes in Poles’ lifestyles and eating behaviours. In subsequent stages, the authors plan to identify more homogeneous groups of individuals while expanding their size so that the results can be translated to a given population of individuals. Indeed, the study group used here was small and its composition was mixed, potentially further weakening the statistical power of the study. Gender and age determine different lifestyles and diets, affecting the number of subgroups and thus the statistical power of tests. The study reported here is an observational pilot study.

## 5. Conclusions

The studies showed that the COVID-19 pandemic had a significant impact on selected elements of the quality of life, such as the body’s efficiency related to the responsibilities of everyday life, ability to work and interpersonal relationships. No significant changes in the frequency of consumption of selected food products were observed. The relationship between the frequency of use of selected drugs and the professional status and ability to work was demonstrated. Taking care of proper eating habits and a hygienic lifestyle is particularly important during a pandemic because they directly affect the health and mental state of people.

## Figures and Tables

**Table 1:** The general characteristics of the studied population.

Sociodemographic Characteristics	Total	
	N = 174	%
Gender		
Woman	142	81.6
Man	32	18.4
Age (years)		
18–25	58	33.3
26–35	46	26.4
36–45	54	31
46–55	10	5.8
≥56	6	3.5
Place of residence		
Village	78	44.8
City < 100,000 inhabitants	52	29.9
City ≥ 100,000 inhabitants	44	25.3
Occupational situation		
Student	54	31
Regular work	50	28.7
Online working	22	12.6
Mixed working	36	20.7
Retired	6	3.5
Unemployed	6	3.5
BMI (kg/m^2^)		
<18.5	20	11.5
18.5–24.9	102	58.6
25.0–29.9	38	21.8
≥30.0	14	8.1

BMI = body mass index; N = number of participants.

**Table 2:** Selected quality of life characteristics [n = 174] [12].

Parameters		Before the COVID-19 Pandemic	During the COVID-19 Pandemic	*p* *
Quality of life	Very poor	0 (0.0%)	4 (2.3%)	1.000
	Poor	10 (5.7%)	8 (4.6%)	
	Neither poor nor good	20 (11.5%)	60 (34.5%)	
	Good	102 (58.6%)	90 (51.7%)	
	Very good	42 (24.1%)	12 (6.9%)	
Health assessment	Very poor	2 (1.1%)	0 (0.0%)	1.000
	Poor	4 (2.3%)	10 (5.7%)	
	Neither poor nor good	42 (24.1%)	64 (36.8%)	
	Good	94 (54.0%)	84 (48.3%)	
	Very good	32 (18.4%)	16 (9.2%)	
Ability to perform your activities of daily living	Very dissatisfied	4 (2.3%)	18 (10.3%)	0.007
	Dissatisfied	16 (9.2%)	50 (28.7%)	
	Neither satisfied nor dissatisfied	36 (20.7%)	60 (34.5%)	
	Satisfied	114 (54.0%)	44 (25.3%)	
	Very satisfied	24 (13.8%)	2 (1.1%)	
Capacity for work	Very dissatisfied	2 (1.1%)	14 (8.0%)	<0.001
	Dissatisfied	6 (3.4%)	34 (19.5%)	
	Neither satisfied nor dissatisfied	46 (26.4%)	76 (43.7%)	
	Satisfied	104 (59.8%)	46 (26.4%)	
	Very satisfied	16 (9.2%)	4 (2.3%)	
Sleep quality assessment	Very dissatisfied	6 (3.4%)	6 (3.4%)	0.999
	Dissatisfied	22 (12.6%)	42 (24.1%)	
	Neither satisfied nor dissatisfied	40 (23.0%)	40 (23.0%)	
	Satisfied	82 (47.1)	68 (39.1%)	
	Very satisfied	24 (13.8%)	18 (10.3%)	
Personal relationships	Very dissatisfied	2 (1.1%)	38 (21.8%)	<0.001
	Dissatisfied	4 (2.3%)	74 (42.5%)	
	Neither satisfied nor dissatisfied	24 (13.8%)	28 (16.1%)	
	Satisfied	90 (51.7%)	26 (14.9%)	
	Very satisfied	54 (31.0%)	8 (4.6%)	

* Chi-square test.

**Table 3:** Frequency of consumption of selected food groups comparing time before and during the COVID-19 pandemic according to BMI and age of respondents (n = 174).

	BMI										Age									
The Frequency of Consumption	Less Frequent			Comparable			More Frequent			*p* *	Less Frequent			Comparable			More Frequent			*p* *
	Me	Q1	Q3	Me	Q1	Q3	Me	Q1	Q3		Me	Q1	Q3	Me	Q1	Q3	Me	Q1	Q3	
Fast food	21.8	20.1	24.0	23.6	20.1	25.9	23.8	20.8	25.8	0.234	35	19	42	35	28	36	19	18	37	0.082
Salty snacks	21.6 ^a^	19.3	22.6	23.8 ^a^	20.2	26.8	23.1	20.2	25.4	0.002	35	18	37	34 ^f^	21	40	27 ^f^	18	36	0.039
Sweets	21.6 ^b^	18.8	22.7	23.6 ^b^	20.2	26.1	23.8	21.0	26.0	0.001	35	18	40	34	23	39.5	34	19	36	0.191
Sweetened sodas	21.6 ^c^	19.9	23.2	23.8 ^c^	20.1	26.1	25.8	36.7	28.1	<0.001	20	18	36	34.5 ^g^	26.5	38.5	35 ^g^	19	36	0.025
Chocolate	21.8 ^d^	19.3	22.7	23.2 ^d^	20.2	25.9	24.5	18.9	27.6	0.026	34	18	37	34	20	39	35	23	36	0.952
Energy drinks	22.6	21.0	23.8	23.8	20.8	25.9	19.6	18.3	27.6	0.052	36 ^h^	27	43	34 ^i^	20	36	18.5 ^h, i^	18	35	0.001
Fruits	21.2	19.5	28.1	23.4	19.0	25.7	22.7	21.9	25.7	0.399	19 ^j, k^	18	34	34.5 ^j^	20	37	35 ^k^	19	40	0.021
Vegetables	22.8	21.3	25.9	23.6	19.5	25.7	22.5	21.0	25.7	0.916	18.5 ^l^	18	27.5	34	20	37	35 ^l^	19	40	0.044
Nuts	21.0	19.2	23.8	23.6	19.5	25.7	23.1	21.6	25.9	0.068	19 ^m, n^	18	26	35 ^m^	20	39	35 ^n^	30	37	<0.001
Cereal products	21.6 ^e^	20.1	22.6	23.8 ^e^	19.5	26.2	21.9	21.0	23.2	0.021	26	18	36	35 ^o^	25	39	20 ^o^	18	35	0.008
Meat and processed products	21.4	20.2	22.6	23.7	20.1	25.9	22.2	20.1	23.8	0.126	19 ^p^	18	40	35 ^p^	25	39	23	19	35	0.003
Dairy product	24.9	18.9	29.9	23.6	20.1	25.8	21.9	20.1	23.1	0.253	26.5	18.5	36.5	35 ^q^	25	39	20 ^q^	19	31	<0.001

a–e, statistically significant difference in BMI value depending on the change in frequency of consumption of selected food groups; f–q, statistically significant difference in age depending on the change in frequency of consumption of selected food groups; * Kruskal–Wallis test. BMI = body mass index; Me = median; Q1 and Q3 = first and third quartiles.

**Table 4:** Frequency of ordering takeaway meals/self-cooking during the COVID-19 pandemic (compared to the time before the COVID-19 pandemic) according to occupational situation and monthly income of respondents (n = 174).

Takeaway Meals							
Parameters		Less Frequent	Comparable		More Frequent		*p* *
Occupational situation	Student	10 (18.5%)	28 (51.9%)		16 (29.6%)		<0.001
	Regular work	12 (24.0%)	24 (48.0%)		14 (28.0%)		
	Online working	6 (27.3%)	12 (54.5%)		4 (18.2%)		
	Mixed working	2 (5.6%)	18 (50.0%)		16 (44.4%)		
	Retired	6 (100%)	0 (0%)		0 (0%)		
	Unemployed	6 (100%)	0 (0%)		0 (0%)		
Monthly income	Decreased	6 (33.3%)	6 (33.3%)		6 (33.3%)		1.000
	Unchanged	36 (26.6%)	68 (50.4%)		31 (23.0%)		
	Increased	0 (0%)	6 (33.3%)		12 (66.7%)		
	Loss of job	0 (0%)	2 (100%)		0 (0%)		
**Self-Cooking Meals**							
**Parameters**		**Definitely No**	**No**	**Hard to Say**	**Yes**	**Definitely Yes**	*p* *
Occupational situation	Student	0 (0%)	8 (14.8%)	12 (22.2%)	24 (44.4%)	10 (18.5%)	0.998
	Regular work	2 (4.0%)	22 (44.0%)	12 (24.0%)	2 (4.0%)	12 (24.0%)	
	Online working	0 (0%)	2 (9.1%)	0 (0%)	6 (27.3%)	14 (63.6%)	
	Mixed working	0 (0%)	10 (27.8%)	10 (27.8%)	8 (22.2%)	8 (22.2%)	
	Retired	0 (0%)	4 (66.7%)	2 (33.3%)	0 (0%)	0 (0%)	
	Unemployed	0 (0%)	2 (33.3%)	0 (0%)	2 (33.3%)	2 (33.3%)	
Monthly income	Decreased	0 (0%)	8 (14.8%)	12 (22.2%)	24 (44.4%)	10 (18.5%)	<0.001
	Unchanged	2 (4.0%)	22 (44.0%)	12 (24.0%)	2 (4.0%)	12 (24.0%)	
	Increased	2 (11.1%)	2 (11.1%)	4 (22.2%)	8 (44.4%)	2 (11.1%)	
	Loss of job	0 (0%)	0 (0%)	0 (0%)	2 (100%)	0 (0%)	

* Chi-square test.

## Data Availability

Data presented in this study are available on request from the corresponding author.

## Supplementary Materials

The following are available online at https://www.mdpi.com/article/10.3390/ijerph182010656/s1, Table S1. Frequency of drug use compared to before and during the COVID-19 pandemic according to selected characteristics defining quality of life and respondents’ current occupations.
