# Supplementary material for: The Impact of the COVID-19 Pandemic on Poles’ Nutritional and Health Behaviour and Quality of Life—A Pilot Study
Source: Int J Environ Res Public Health. 2021 Oct 12;18(20):10656. doi: 10.3390/ijerph182010656 (PMC8535850; doi:10.3390/ijerph182010656)
Supplement: Supplementary file 1 [file ijerph-18-10656-s001.zip › ijerph-1375830-supplementary.pdf]

Supplementary tables

Table S1. Frequency of stimulant use compared to before and during the COVID-19 pandemic according to selected characteristics defining quality of life and respondents' current occupations.

Parameters		Cigarettes						Alcohol						Coffee					
		Definitely no	No	Hard to say	Yes	Definitely yes	p*	Definitely no	No	Hard to say	Yes	Definitely yes	p*	Definitely no	No	Hard to say	Yes	Definitely yes	p*
Occupational situation	Student	18 (33.3%)	14 (25.9%)	8 (14.8%)	6 (11.1%)	8 (14.8%)	<0.001	6 (11.1%)	24 (44.4%)	14 (25.9%)	6 (11.1%)	4 (7.4%)	<0.001	10 (18.5%)	22 (40.7%)	6 (11.1%)	6 (11.1%)	10 (18.5%)	<0.001
	Regular work	10 (20.0%)	34 (68%)	2 (8%)	4 (8%)	0 (0%)		2 (4%)	28 (56%)	6 (12%)	12 (24%)	2 (4%)		0 (0%)	28 (56%)	8 (16%)	10 (20%)	4 (8%)	
	Online work	6 (27.3%)	10 (45.4%)	2 (9.1%)	2 (9.1%)	2 (9.1%)		8 (36.4%)	4 (18.2%)	4 (18.2%)	4 (18.2%)	2 (9.1%)		2 (9.1%)	6 (27.3%)	2 (9.1%)	0 (0%)	12 (54.5%)	
	Mixed work	14 (39.9%)	10 (27.8%)	4 (11.1%)	4 (11.1%)	4 (11.1%)		14 (38.9%)	10 (27.8%)	0 (0%)	10 (27.8%)	2 (5.6%)		14 (38.9%)	12 (33.3%)	2 (5.6%)	8 (22.2%)	0 (0%)	
	Retired	6 (100%)	0 (0%)	0 (0%)	0 (0%)	0 (0%)		2 (33.3%)	4 (66.7%)	0 (0%)	0 (0%)	0 (0%)		0 (0%)	6 (100%)	0 (0%)	0 (0%)	0 (0%)	
	Unemployed	0 (0%)	4 (66.7%)	0 (0%)	2 (33.3%)	0 (0%)		0 (0%)	6 (100%)	0 (0%)	0 (0%)	0 (0%)		0 (0%)	6 (100%)	0 (0%)	0 (0%)	0 (0%)	
Quality of life	Very poor	2 (50%)	0 (0%)	2 (50%)	0 (0%)	0 (0%)	0.339	0 (0%)	0 (0%)	2 (50%)	2 (50%)	0 (0%)	0.695	0 (0%)	4 (100%)	0 (0%)	0 (0%)	0 (0%)	0.997
	Poor	4 (50%)	2 (25%)	0 (0%)	2 (25%)	0 (0%)		4 (50%)	0 (0%)	0 (0%)	4 (50%)	0 (0%)		4 (50%)	2 (25%)	0 (0%)	2 (25%)	0 (0%)	

	Neither poor nor good	18 (30%)	22 (36.7%)	8 (13.3%)	8 (13.3%)	4 (6.7%)		8 (13.3%)	34 (56.7%)	8 (13.3%)	10 (16.7%)	0 (0%)		14 (23.3%)	26 (43.3%)	6 (10%)	10 (16.7%)	4 (6.7%)	
	Good	26 (28.9%)	46 (51.1%)	4 (4.4%)	4 (4.4%)	10 (11.1%)		18 (20%)	34 (37.8%)	14 (15.6%)	14 (15.6%)	10 (11.1%)		8 (8.9%)	42 (46.7%)	10 (11.1%)	10 (11.1%)	20 (22.2%)	
	Very good	4 (33.3%)	2 (16.7%)	2 (16.7%)	4 (33.3%)	0 (0%)		2 (16.7%)	8 (66.7%)	0 (0%)	2 (16.7%)	0 (0%)		0 (0%)	6 (50%)	2 (16.7%)	2 (16.7%)	2 (16.7%)	
Health	Very bad	0 (0%)	0 (0%)	0 (0%)	0 (0%)	0 (0%)		0 (0%)	0 (0%)	0 (0%)	0 (0%)	0 (0%)		0 (0%)	0 (0%)	0 (0%)	0 (0%)	0 (0%)	
	Bad	6 (60%)	0 (0%)	2 (20%)	0 (0%)	2 (20%)		4 (40%)	0 (0%)	2 (20%)	4 (40%)	0 (0%)		0 (0%)	4 (40%)	2 (20%)	2 (20%)	2 (20%)	
	Neither satisfied nor dissatisfied	12 (18.7%)	34 (53.1%)	4 (6.2%)	10 (15.6%)	4 (6.2%)	0.243	6 (9.4%)	28 (43.8%)	8 (12.5%)	18 (28.1%)	4 (6.3%)	0.853	12 (18.8%)	32 (50%)	4 (6.3%)	10 (15.6%)	6 (9.4%)	0.931
	Good	26 (30.9%)	34 (40.5%)	10 (11.9%)	6 (7.1%))	8 (9.5%)		20 (23.8%)	38 (45.2%)	12 (14.3%)	8 (9.5%)	6 (7.1%)		10 (11.9%)	34 (40.5%)	12 (14.3%)	12 (14.3%)	16 (19.1%)	
	Very good	10 (62.5%)	4 (25%)	0 (0%)	2 (12.5%)	0 (0%)		2 (12.5%)	10 (62.5%)	2 (12.5%)	2 (12.5%)	0 (0%)		4 (25%)	10 (62.5%)	0 (0%)	0 (0%)	2 (12.5%)	
Ability to perform activities of daily living	Very dissatisfied	6 (33.3%)	4 (22.2%)	4 (22.2%)	4 (22.2%)	0 (0%)		2 (11.1%)	4 (22.2%)	4 (22.2%)	8 (44%).	0 (0%)		4 (22.2%)	1 (55.6%)0	2 (11.1%)	2 (11.1%)	0 (0%)	
	Dissatisfied	16 (32%)	18 (36%)	2 (4%)	8 (16%)	6 (12%)	0.042	16 (32%)	12 (24%)	6 (12%)	16 (32%)	0 (0%)	0.023	10 (20%)	8 (16%)	12 (24%)	10 (20%)	10 (20%)	0.179
	Neither satisfied nor	20 (33.3%)	26 (43.3%)	6 (10%)	2 (3.3%)	6 (10%)		6 (10%)	36 (60%)	8 (13.3%)	2 (3.3%)	8 (13.3%)		8 (13.3%)	34 (56.7%)	2 (3.3%)	6 (10%)	10 (16.7%)	

	dissatisfied																		
	Satisfied	10 (27.3%)	24 (54.6%)	4 (9.1%)	4 (9.1%)	2 (4.6%)		6 (13.6%)	24 (54.6%)	6 (13.6%)	6 3.6%)	2 (4.6%)		4 (4.6%)	26 (59.1%)	2 (4.6%)	6 (13.6%)	6 (13.6%)	
	Very satisfied	2 (100%)	0 (0%)	0 (0%)	0 (0%)	0 (0%)		2 (100%)	0 (0%)	0 (0%)	0 (0%)	0 (0%)		0 (0%)	2(100%)	0 (0%)	0 (0%)	0 (0%)	
Capacity for work	Very dissatisfied	4 (28.6%)	4 (28.6%)	6 (42.9%)	0 (0%)	0 (0%)	<0,001	0 (0%)	2 (14.3%)	2 (14.3%)	8 (57.1%)	2 (14.3%)	<0,001	0 (0%)	8 (27.1%)	0 (0%)	2 (14.3%)	4 (28.6%)	0,002
	Dissatisfied	8 (23.5%)	14 (41.2%)	2 (5.9%)	4 (11.8%)	6 (17.6%)		6 (17.6%)	12 (35.3%)	6 (17.6%)	8 (23.5%)	2 (5.9%)		8 (23.5%)	12 (35.3%)	8 (23.5%)	0 (0%)	6 (17.7%)	
	Neither satisfied nor dissatisfied	24 (31.6%)	36 (47.4%)	6 (7.9%)	6 (7.9%)	4 (5.3%)		14 (18.4%)	36 (47.4%)	14 (18.4%)	10 (13.2%)	2 (2.6%)		10 (13.2%)	36 (47.4%)	4 (5.3%)	16 (21.1%)	10 (13.2%)	
	Satisfied	18 (39.1%)	16 (34.8%)	2 (4.4%)	6 (13%)	4 (8.7%)		10 (21.7%)	24 (52.2%)	2 (4.3%)	6 (13%)	4 (8.7%0		8 (17.4%)	26 (56.5%)	4 (8.7%)	2 (4.3%)	6 (13%)	
	Very satisfied	0 (0%)	2 (50%)	0 (0%)	2 (50%)	0 (0%)		2 (50%)	2 (50%)	0 (0%)	0 (0%)	0 (0%)		2 (50%)	2 (50%)	0 (0%)	0 (0%)	0 (0%)	
Sleep quality	Very dissatisfied	2 (33.3%)	2 (33.3%)	0 (0%)	0 (0%)	2 (33.3%)	0.102	2 (33.3%)	2 (33.3%)	0 (0%)	2 (33.3%)	0 (0%)	0.459	2 (33.3%)	2 (33.3%)	0 (0%)	2 (33.3%)	0 (0%)	0.037
	Dissatisfied	18 (42.9%)	14 (33.3%)	2 (4.8%)	4 (9.5%)	4 (9.5%)		10 (23.8%)	8 (19.1%)	12 (25.6%)	10 (23.8%)	2 (4.8%)		6 (14.3%)	12 (28.6%)	4 (9.5%)	10 (23.8%)	10 (23.8%)	
	Neither satisfied nor dissatisfied	8 (20%)	20 (50%)	8 (20%)	4 (10%)	0 (0%)		8 (20%)	20 (50%)	0 (0%)	8 (20%)	4 (10%)		4 (10%)	22 (55%)	0 (0%)	4 (10%)	10 (25%)	

	Satisfied	20 (29.4%)	32 (47.1%)	4 (5.9%)	6 (8.8%)	6 (8.8%)		8 (11.8%)	38 (55.9%)	8 (11.8%)	12 (17.7%)	2 (2.9%)		12 (17.7%)	38 (55.9%)	8 (11.8%)	6 (8.8%)	4 (5.9%)	
	Very satisfied	6 (33.3%)	4 (22.2%)	2 (11.1%)	4 (22.2%)	2 (11.1%)		4 (22.2%)	8 (44.4%)	4 (22.2%)	0 (0%)	2 (11.1%)		2 (11.1%)	6 (33.3%)	6 (33.3%)	2 (11.1%)	2 (11.1%)	
Negative feelings	Never	6 (42.9%)	2 (14.3%)	0 (0%)	2 (14.3%)	4 (28.6%)		2 (14.3%)	4 (28.6%)	4 (28.6%)	2 (14.3%)	2 (14.3%)		4 (25.6%)	6 (42.9%)	2 (14.3%)	0 (0%)	2 (14.3%)	
	Infrequently	18 (25.7%)	36 (51.4%)	6 (8.6%)	8 (11.4%)	2 (2.9%)		12 (17.1%)	40 (57.1%)	6 (8.6%)	10 (14.3%)	2 (2.9%)		10 (14.3%)	36 (51.4%)	6 (8.6%)	12 (17.1%)	6 (8.6%)	
	Sometimes	24 (35.3%)	26 (38.2%)	6 (8.8%)	6 (8.8%)	6 (8.8%)	0.124	16 (23.5%)	24 (35.3%)	10 (14.7%)	12 (17.7%)	6 (8.8%)	0.699	8 (11.8%)	26 (38.2%)	6 (8.8%)	10 (14.7%)	18 (26.5%)	0.548
	Frequently	6 (27.3%)	8 (36.4%)	4 (18.2%)	2 (18.2%)	2 (9.1%)		2 (9.1%)	8 (36.4%)	4 (18.2%)	8 (36.4%)	0 (0%)		4 (18.2%)	12 (54.6%)	4 (18.2%)	2 (9.1%)	0(0%)	
	Always	0 (0%)	0 (0%)	0 (0%)	0 (0%)	0 (0%)		0 (0%)	0 (0%)	0 (0%)	0 (0%)	0 (0%)		0(0%)	0 (0%)	0 (0%)	0 (0%)	0 (0%)	

*Chi- square test

Table S2

Ladies and gentlemen,

We kindly ask you to fill in the following questionnaire, prepared in the Department of Human Nutrition at Wrocław University of Environmental and Life Sciences.

The questionnaire is completely anonymous and its results will be used only for scientific work, so please fill it in independently and conscientiously.

This assessment asks how you feel about your quality of life, health, or other areas of your life. Please answer all the questions. If you are unsure about which response to give to a question, please choose the one that appears most appropriate. This can often be your first response.

Thank you in advance for your time and reliable answers.

*Required

1. Gender *

Select only one answer.

☐ female

☐ male

2. Age *

____

3. Accommodation*

Select only one answer.

- ☐ city with more than 500 thousand inhabitants
- ☐ city with 100,000 - 500,000 inhabitants
- ☐ city with less than 100 thousand inhabitants
- ☐ village

4. Growth [cm] *

____

5. Current weight [kg] *

____

6. Body weight before the pandemic COVID-19 [kg] *

____

7. Which of the following statements best describes you (last 12 months) *

Select only one answer.

☐ I work online

☐ I work stationary

☐ My work mode is mixed: part online, part stationary

☐ I am a student

☐ I am unemployed

☐ I am retired

8. How would you rate your current quality of life?*

Select only one answer.

☐ very poor

☐ poor

☐ neither poor nor good

☐ good

☐ very good

9. How would you rate your quality of life before pandemic COVID-19? *

Select only one answer.

☐ very poor
☐ poor
☐ neither poor nor good
☐ good
☐ very good

10. How do you assess your current health?*

Select only one answer.

☐ very poor
☐ poor
☐ neither poor nor good
☐ good
☐ very good

11. How do you assess your health before pandemic COVID-19? *

Select only one answer.

- ☐ very poor
- ☐ poor
- ☐ neither poor nor good
- ☐ good
- ☐ very good

The following questions refer to any “daily living activities” that you do. Daily living activities here means any majornactivity that you do. This includes voluntary work, studying full-time, taking care of the home, taking care of children, shopping, hobby.

12. How satisfied are you with your current ability to perform your daily living activities? *

Select only one answer.

- ☐ very dissatisfied
- ☐ dissatisfied
- ☐ neither satisfied nor dissatisfied
- ☐ satisfied
- ☐ very satisfied

13. How satisfied are you with your ability to perform your daily living activities before pandemic COVID-19? *

Select only one answer.

☐ very dissatisfied

☐ dissatisfied

☐ neither satisfied nor dissatisfied

☐ satisfied

☐ very satisfied

The following questions refer to any “work” that you do. Work here means any major paid work that you do.

14. How satisfied are you with your current capacity for work? *

Select only one answer.

☐ very dissatisfied

☐ dissatisfied

☐ neither satisfied nor dissatisfied

☐ satisfied

☐ very satisfied

15. How satisfied are you with your capacity for work before pandemic COVID-19? *

Select only one answer.

- ☐ very dissatisfied
- ☐ dissatisfied
- ☐ neither satisfied nor dissatisfied
- ☐ satisfied
- ☐ very satisfied

16. How satisfied are you with your sleep? *

Select only one answer.

- ☐ very dissatisfied
- ☐ dissatisfied
- ☐ neither satisfied nor dissatisfied
- ☐ satisfied
- ☐ very satisfied

17. How satisfied are you with your sleep before pandemic COVID-19? *

Select only one answer.

- ☐ very dissatisfied
- ☐ dissatisfied
- ☐ neither satisfied nor dissatisfied
- ☐ satisfied
- ☐ very satisfied

The following questions ask you to say how satisfied, happy or good you have felt about

personal relationship. For example contact with you friends, contact with your work colleagues, contact with your family in other cities or countries.

18. How satisfied are you with your current personal relationships? *

Select only one answer.

- ☐ very dissatisfied
- ☐ dissatisfied
- ☐ neither satisfied nor dissatisfied
- ☐ satisfied
- ☐ very satisfied

19. How satisfied are you with your personal relationships before pandemic COVID-19? *

Select only one answer.

- ☐ very dissatisfied
- ☐ dissatisfied
- ☐ neither satisfied nor dissatisfied
- ☐ satisfied
- ☐ very satisfied

20. Did your monthly income change during the COVID-19 pandemic compared to before the COVID-19 pandemic? *

Select only one answer.

- ☐ decreased
- ☐ unchanged
- ☐ increased
- ☐ loss of job

21. How has the frequency of your ordering take-out meals changed during the COVID-19 pandemic (compared to before the COVID-19 pandemic)?*

Select only one answer.

☐ higher than before the pandemic
☐ comparable to pre-pandemic levels
☐ lower than before the pandemic

22. How did the frequency of your consumption of the following food groups change during the COVID-19 pandemic (compared to before the COVID-19 pandemic) *

Select only one answer in a row.

	less	comparable	more
**fast food**	⬭	⬭	⬭
**salty snacks**	⬭	⬭	⬭
**sweets**	⬭	⬭	⬭

**sweetened sodas**	○	○	○
**chocolate**	○	○	○
**energy drinks**	○	○	○
**fruits**	○	○	○
**vegetables**	○	○	○
**nuts**	○	○	○
**cereal products**	○	○	○

meat and processed products

dairy products

23. Did you cook on your own more often during the COVID-19 pandemic than before the COVID-19 pandemic?*

Select only one answer.

- ☐ definitely no
- ☐ no
- ☐ hard to say
- ☐ yes
- ☐ definately yes

25. Do you smoke cigarettes? *

Select only one answer.

- ☐ yes

- ☐ no

26. If yes, have you observed a tendency to smoke more frequently during the COVID-19 pandemic (compared to before the COVID-19 pandemic)?*

Select only one answer.

- ☐ definitely no
- ☐ no
- ☐ hard to say
- ☐ yes
- ☐ definately yes

27. Did you drink more alcoholic drinks during the COVID-19 pandemic (compared to before the COVID-19 pandemic)? *

Select only one answer.

- ☐ definitely no
- ☐ no
- ☐ hard to say
- ☐ yes
- ☐ definately yes

28. Did you drink more coffee during the COVID-19 pandemic (compared to before the COVID-19 pandemic)?

Select only one answer.

- ☐ definitely no
- ☐ no
- ☐ hard to say
- ☐ yes
- ☐ definately yes

THANK YOU FOR YOUR HELP
